# Shoulder instability and associated shoulder injuries in patients with epilepsy

**DOI:** 10.1186/s12891-026-10180-w

**Published:** 2026-07-06

**Authors:** Alp Paksoy, Henry Gebauer, Samuel Kemoh Sesay, Soraya Bahlawane, Agahan Hayta, Maximilian Hinz, David Alexander Back, Rony-Orijit Dey Hazra, Poroshista Knauer, Doruk Akgün

**Affiliations:** Center for Musculoskeletal Surgery, Charité-Universitätsmedizin Berlin, Corporate Member of Freie Universität Berlin and Humboldt-Universität zu Berlin, Augustenburger Pl. 1, Berlin, 13353 Germany

**Keywords:** Epileptic seizures, Shoulder dislocation, Fracture dislocation, Instability, Epilepsy, Gamma angle, Hill-Sachs lesion, Reverse Hill-Sachs lesion, Pico method

## Abstract

**Background:**

The epidemiology and characteristics of shoulder dislocations in the context of epileptic seizures, as well as treatment recommendations, have so far been not described in detail in the literature. The aim of this retrospective study is to document the epidemiological characteristics and treatment options of shoulder instabilities occurring during epileptic seizures, as well as to quantify associated injuries.

**Methods:**

As part of a retrospective analysis, 72 shoulders in 56 patients at our clinic were evaluated who sustained shoulder dislocation during an epileptic seizure. An analysis of the epidemiology and key characteristics was conducted. Sectional imaging including computer tomography scans and/or magnetic resonance imaging of all patients were independently analyzed at different time points by two raters (A.P. and H.G.) using OsiriX™ (Geneva, Switzerland) for glenoid defects as well as the Hill-Sachs lesion (HSL) and the reverse HSL (RHSL). When present, surgical treatment modalities were analyzed.

**Results:**

The cohort had a mean age of 37 ± 16 years; 12 were female (21%) and 44 were male (79%). A total of 72 shoulder dislocations were identified: 60% anterior (43/72), 30% posterior (22/72), and 10% bidirectional (7/72); 51% were first-time dislocations (37/72) and 49% recurrent (35/72). Radiological imaging suitable for quantitative defect analysis was available for 47 shoulders, including 30 anterior and 17 posterior dislocations.

The anterior glenoid defect size of patients with anterior dislocation averaged 11% (± 8%) and the mean posterior glenoid defect size of patients with posterior dislocation was 7% (± 6%). 16 of the 30 anterior dislocations and three of the 17 posterior shoulder dislocations were recurrent. Significant differences between first-time and recurrent dislocations were found in HSL width (13 mm vs. 15 mm; p = 0.008), HSL length (24 mm vs. 29 mm; p < 0.001), posterior glenoid defect size (5% vs. 13%; p = 0.04), and reverse HSL γ-angle (114° vs. 84°; p = 0.003).

Fractures occurred in 46.4% of patients (26/56), all involving the proximal humerus, three patients also sustained a glenoid fracture. 62.5% of injuries were treated surgically (45/72). Surgical techniques included soft tissue stabilization (26.7%; 12/45), bony augmentation (24.4%; 11/45), arthroplasty (4.4%; 2/45), and humeral open reduction and internal fixation (44.4%; 20/45).

**Conclusion:**

In patients with epilepsy, shoulder dislocations predominantly occur during generalized seizures, with anteroinferior dislocations being the most frequent, while posterior dislocations are significantly more prevalent compared to the general population. Recurrent dislocations were associated with significantly larger humeral and posterior glenoid defects, indicating progressive bone loss. Approximately half of all injuries involved proximal humerus fractures, with more than half requiring surgical intervention.

**Level of Evidence:**

IV.

## Introduction

Musculoskeletal injuries are a recognized yet frequently underappreciated complication of epileptic seizures. Approximately 33% of seizure-related fractures involve the shoulder, making it the most commonly affected joint, followed by thoracic and lumbar vertebrae (29%), the skull and jaw (8%), and the femoral neck (6%) [1–3]. Seizure-related shoulder trauma may result in a wide range of injuries, including fractures of the humerus, scapula, or clavicle, as well as acute or recurrent dislocations, rotator cuff tears, or complex combinations of these conditions [1–3]. The risk of injury varies depending on seizure type, with generalized tonic–clonic and myoclonic seizures carrying the highest risk, while both primarily and secondarily generalized seizures have been associated with fracture occurrence [4]. These injuries are frequently severe, often requiring surgical intervention, yet their epidemiology and specific lesion patterns remain insufficiently reported.

Despite the clinical relevance of these injuries, the predominant direction of shoulder instability in this patient population remains unclear, as current literature offers conflicting reports [5–8]. Notably, posterior shoulder dislocations—typically rare in the general population—appear with increased frequency among patients with epileptic seizures [9]. Moreover, the extent of seizure-related bone loss, such as glenoid defects and Hill-Sachs lesions (HSLs) or reverse HSL (RHSL), and their relationship to recurrent instability events is poorly understood.

To date, there is no established consensus or standardized treatment algorithm for managing seizure-related shoulder instability [10], and outcomes are often compromised by high recurrence rates and associated fractures [1, 6, 11]. Given the unique biomechanical and neurological context of these injuries, further characterization is needed. In particular, detailed information regarding instability patterns, associated bone defects, and treatment strategies remains limited despite their potential relevance for clinical decision-making. The present study aimed to analyze the epidemiology of epilepsy-related shoulder injuries in a relatively large patient cohort and to provide insights into specific lesion patterns, as well as to quantify all defects, and their respective treatment strategies. It is hypothesized that epilepsy-related shoulder dislocations are predominantly anteroinferior but show a higher proportion of posterior dislocations than in the general population, are associated with more frequent and extensive glenoid and humeral defects in recurrent cases regardless of dislocation direction, and have an increased incidence of fractures necessitating operative treatment.

## Materials and methods

This retrospective exploratory study aimed to investigate the epidemiology, characteristics, and treatment of shoulder dislocations associated with seizures. Patients treated at our orthopedic center between 2012 and 2023 with the diagnosis of both epilepsy and shoulder dislocation were included. The primary objective was to characterize the pattern of dislocation, associated injuries, treatment strategies, and radiological findings in this specific patient population. The vote of the responsible local ethics committee was obtained (EA1/157/24).

### Study cohort

Basic demographic data such as gender, date of birth, diagnosis, and comorbidities were collected. Additionally, epilepsy type (classified as generalized or focal based on patient history), potential trigger factors, epilepsy status (first-time seizure vs. known epilepsy, derived from anamnesis or multiple patient stays), and ongoing anticonvulsant therapy were considered. For the analysis of shoulder dislocations, the direction of instability, the frequency of dislocations, the affected side, and the age at the time of dislocation were documented. Recurrent instability was considered present if at least two dislocation episodes of the same shoulder were documented. The treatment was classified as either conservative or surgical, with the type of procedure - open reduction and internal fixation (ORIF), soft tissue stabilization, bony augmentation (e.g. iliac crest bone graft transfer or Latarjet procedure) or arthroplasty - being recorded. At our institution, soft tissue stabilization is generally performed in patients with minimal or no glenoid bone loss, whereas bony stabilization procedures are preferred in cases with glenoid bone loss exceeding 15–20%.

### Radiological evaluation

**Fig. 1:**
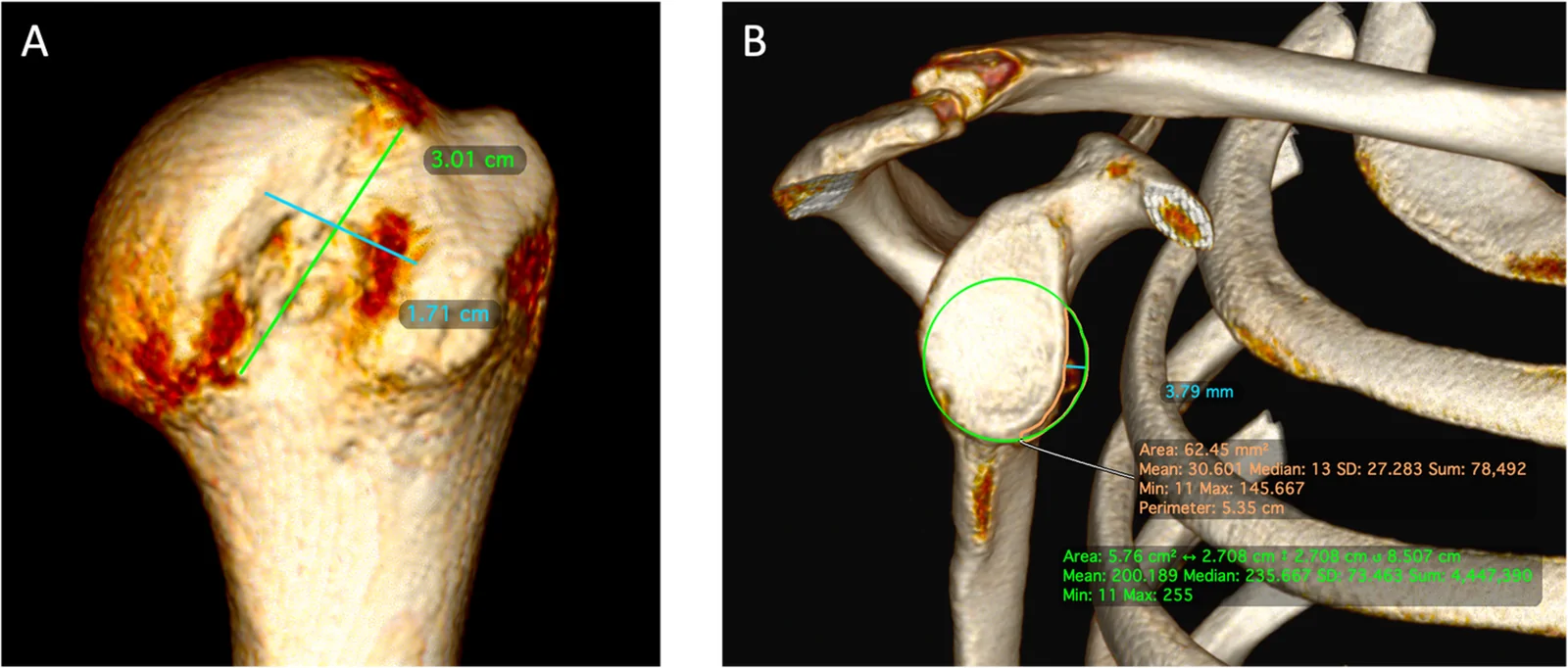
Radiological evaluation of shoulder lesions in patients with seizure-related dislocations performed in OsiriX™. **A** Example of a Hill-Sachs lesion (HSL) with measurements of length (green line, 3.01 cm) and width (blue line, 1.71 cm). **B** Example of an anterior glenoid defect assessed in the en face view using the PICO method; the area, perimeter, and other quantitative parameters are displayed

Radiological diagnostics, if available, were evaluated, including rotator cuff lesions, capsulolabral lesions, fractures, HSLs and Bankart lesions. The computer tomography (CT) scans (or magnetic resonance imaging (MRI), if CT was not available) of all patients were evaluated independently by two raters (A.P. and H.G.) at different time points using OsiriX™ (Geneva, Switzerland). The length, width, and depth of the HSL were measured in millimeters [12–14] (Fig. 1, A). The glenoid defect was measured in all patients using the PICO method in the en face view of CT scans or MRI imaging [12, 15, 16] (Fig. 1, B). The location of the RHSL was assessed using the angle method according to Moroder et al. [17].

### Statistical analysis

The results were analyzed descriptively, the radiological measurements were analyzed with central tendency such as means and medians, as well as measures of dispersion including standard deviation being reported. The Shapiro Wilk test in combination with histogram was used to examine radiological measurements for a normal distribution. The unpaired t-test (for normally distributed data) and the Mann-Whitney-U test (for non-normally distributed data) were utilized to compare continuous variables between first time dislocated shoulders with the recurrent dislocated shoulders. For Mann-Whitney U tests, effect size is reported as r = Z / √N. For normally distributed variables effect size is reported as Cohen’s d. Additionally, intra-class correlation coefficients (ICCs) with a 95% confidence interval were calculated for all measurements conducted twice by two raters. According to Landis and Koch, an ICC below 0.20 indicates slight agreement, values between 0.21 and 0.40 suggest fair agreement, 0.41 to 0.60 represent moderate agreement, 0.61 to 0.80 indicate substantial agreement, and values above 0.81 correspond to almost perfect agreement [18]. Following the reliability assessment, the ratings from both evaluators were averaged for subsequent analyses. The statistical analysis was performed using IBM SPSS Statistics 29.0 software (IBM, Armonk, NY, USA).

## Results

56 patients with shoulder dislocations due to an epileptic seizure were identified, the average age was 36.6 ± 16 years at the time of dislocation. Of the 56 patients, 12 were female (21.4%) and 44 were male (78.6%).

Most patients (82.1%) experienced generalized seizures, 12.5% had focal seizures, 5.4% were not reported. 35.7% of the patients were able to identify trigger factors (20/56), of which five were alcohol-related, three were drug-related, three were tumor-related, two triggered by sleep deprivation, two by infection, two in the context of hyponatremia, one pregnancy-related, one following a medial infarction, and one by psychological stress. 33.9% of the patients had their first epileptic seizure (19/56). 25 patients had a seizure while on existing anticonvulsant therapy (44.6%; 25/56).

A total of 72 different shoulders with instabilities were identified in 56 patients. Of these, 43 were anterior (59.7%), 22 were posterior (30.6%), and seven were bidirectional, i.e., involving both anterior and posterior (9.7%). 51.4% of the shoulder dislocations were first time dislocations (37/72), 48.6% were recurrent dislocations (35/72).

### Radiological evaluation

Only 30 of the 41 anterior and 17 of the 24 posterior shoulder instabilities had imaging available for measuring the glenoid defect, HSL, and RHSL. 16 of the 30 anterior dislocations were recurrent shoulder dislocations and three of the 17 posterior dislocations were recurrent.

Among anterior dislocations, the average glenoid defect size was 11% (± 8%), with no significant difference between first-time (11% ± 9%) and recurrent (10% ± 6%) cases (*p* = 1.0). In contrast, posterior dislocations showed a significantly larger glenoid defect in recurrent cases (13% ± 11%) compared to first-time dislocations (5% ± 3%, *p* = 0.04).

Regarding HSL, recurrent anterior dislocations were associated with significantly greater HSL width (15 mm ± 3 mm vs. 13 mm ± 2 mm, *p* = 0.008) and HSL length (29 mm ± 4 mm vs. 24 mm ± 3 mm, *p* < 0.001). No significant difference was observed in HSL depth between groups (8 mm ± 2 mm vs. 7 mm ± 2 mm, *p* = 0.172). In RHSLs among posterior dislocations, the γ-angle was significantly lower in recurrent cases (84° ± 5°) compared to first-time dislocations (114° ± 23°, *p* = 0.003) (Table 1).

**Table 1:** Radiological measurements of osseous defects and lesion morphology in the shoulder joint, divided in first and recurrent dislocations

Radiological Measurement	Study Cohort	First Time Dislocation	Recurrent Dislocations	*p*-value	Test Statistic	Effect Size
Glenoid defect size (anterior)	11% (± 8%) *n* = 30	11% (± 9%) *n* = 14	10% (± 6%) *n* = 16	1.0	Z = 0.000	*r* = 0.00 no effect
Glenoid defect size (posterior)	7% (± 6%) *n* = 17	5% (± 3%) *n* = 14	13% (± 11%) *n* = 3	0.04*	Z = -2.019	*r* = -0.24; small-medum effect
HSL width	14 mm (± 3 mm) *n* = 30	13 mm (± 2 mm) *n* = 14	15 mm (± 3 mm) *n* = 16	0.008*	t(45.42) = -2.793	Cohen’s d = -0.60; medium effect
HSL depth	8 mm (± 2 mm) *n* = 30	7 mm (± 2 mm) *n* = 14	8 mm (± 2 mm) *n* = 16	0.172	Z = -1.367	*r* = -0.17 small effect
HSL length	28 mm (± 5 mm) *n* = 30	24 mm (± 3 mm) *n* = 14	29 mm (± 4 mm) *n* = 16	< 0.001*	t(66) = -4.250	Cohen’s d = -1.22; large effect
γ-angle of RSHL	99° (± 22°) *n* = 17	114° (± 23°) *n* = 14	84° (± 5°) *n* = 3	0.003*	Z = -2.836	*r* = -0.71; very large effect

Values are presented as mean ± standard deviation

*HSL* Hill-Sachs lesion, *RHSL* Reverse Hill-Sachs lesion, *γ-angle* measurement of lesion orientation

Statistically significant p-values are marked with an asterisk (*)

All measurements performed showed nearly perfect agreement between the two raters (Table 2)

**Table 2:** Inter- and intrarater reliability for radiological measurements of shoulder lesions, reported as intraclass correlation coefficients (ICC) with 95% confidence intervals (CI)

Measurement	Interrater reliability			Intrarater reliability (A.*P*.)			Intrarater reliability (H.G.)		
	ICC	CI-	CI+	ICC	CI-	CI+	ICC	CI-	CI+
Glenoid defect size anterior	0.917	0.855	0.957	0.925	0.844	0.964	0.847	0.681	0.927
Glenoid defect size posterior	0.960	0.918	0.984	0.956	0.878	0.984	0.960	0.888	0.985
HSL width	0.892	0.775	0.956	0.864	0.572	0.953	0.954	0.873	0.983
HSL length	0.938	0.870	0.975	0.857	0.599	0.948	0.970	0.919	0.989
HSL depth	0.960	0.916	0.984	0.949	0.841	0.982	0.969	0.915	0.989
γ-angle of RSHL	0.971	0.868	0.998	0.876	-3.759	0.992	0.994	0.942	1.000

Measurements were performed by two raters (A.P. and H.G.) and compared across repeated sessions

*HSL* Hill-Sachs lesion, *γ-angle* measurement of lesion orientation

Fractures occurred in 26 of 56 patients (46.4%) in the overall study cohort. All fractures involved the proximal humerus, and three patients additionally sustained a glenoid fracture.

Overall, 62.5% of all shoulder injuries were treated surgically (45/72). Soft tissue stabilization was performed in 26.7% of surgically treated shoulders (12/45). Bony augmentation procedures accounted for 24.4% (11/45) and included eight iliac crest bone graft augmentations, one Latarjet procedure, one cerclage with a spina scapulae graft, and one anterior glenoid reconstruction with biocompression screws. Shoulder arthroplasty was performed in 4.4% of cases (2/45), with both patients undergoing hemiarthroplasty using the Eclipse system (Arthrex Inc., Naples, FL, USA). ORIF was performed in 44.4% of surgically treated shoulders (20/45). Among these, three patients required additional glenoid fixation, including two glenoid reconstructions with biocompression screws and one fixation using the independent double-row technique described by Moroder et al. [19].

## Discussion

The present study examined the epidemiology and specific lesion patterns with bony defects in a large cohort of patients with seizure-related shoulder injuries, highlighting the relatively high incidence of posterior dislocations in this patient group compared to the general population. Most patients experienced generalized seizures and almost the half of the patients had a seizure while on existing anticonvulsant therapy. The half of all shoulder injuries were accompanied by fractures, many of which necessitated surgical intervention.

The most frequently reported direction of instability in patients with epilepsy varies between studies. Although literature often links epileptic seizures with posterior shoulder dislocations [8, 20–22], the prevailing direction of instability in the aforementioned patient cohorts was anterior [1, 6, 20]. In the present study, anterior shoulder instability was confirmed as the dominant direction among patients with seizures (60%); nevertheless, a substantial proportion (30%) experienced posterior dislocations within this selected population. Notably, the incidence of a rare injury such as posterior shoulder dislocation was higher in the present study compared to literature data from populations without epilepsy [1, 9, 20, 23, 24].

An increased incidence of fractures among epilepsy patients has been well documented, which was also the case in the present study [25–27]. This highlights the considerable mechanical forces acting on the shoulder joint during seizures. Epileptic seizures can result in unprotected falls, leading to fractures, or the uncontrolled and imbalanced muscle contractions that occur during seizures can generate extreme forces on the musculoskeletal system, potentially causing joint dislocations or fracture-dislocations in both directions [1, 28].

To our knowledge, this is one of the first studies to evaluate glenoid and humeral head defect sizes with the forementioned methodology specifically in patients with shoulder dislocations associated with epileptic seizure. Interestingly, the mean glenoid defect sizes observed in our study—11% for anterior and 7% for posterior dislocations—were lower than expected, which often links seizure-related shoulder instability with more severe bone loss due to frequent and forceful recurrent dislocations [5, 29]. However, further analysis revealed significant differences between first-time and recurrent dislocations. In anterior instability, recurrent cases demonstrated significantly larger HSL in width (*p* = 0.008) and length (*p* < 0.001), indicating cumulative damage to the humeral head. The same findings were also reported by Bige et al., who analyzed 50 epileptic and non-epileptic patients with anterior recurrent shoulder instability [30]. They demonstrated that HSLs are larger and deeper in patients with epilepsy and shoulder instability than in non-epileptic patients with shoulder instability (depth: 22% vs. 9%, *p* < 0.001; width: 43% vs. 28%, *p* = 0.003) [30]. For posterior dislocations, glenoid defect size was significantly greater in recurrent cases (13% vs. 5%, *p* = 0.04). These findings support the notion that even if initial imaging may reveal only modest bone loss, repeated instability events can lead to progressive and clinically relevant bony defects. However, not all radiological parameters differed significantly between first-time and recurrent dislocations. In particular, anterior glenoid defect size was comparable between groups (11% ± 9% vs. 10% ± 6%; *p* = 1.0), and no significant difference was observed for HSL depth (7 ± 2 mm vs. 8 ± 2 mm; *p* = 0.172). This may indicate that recurrent instability in patients with epilepsy does not uniformly affect all osseous defect characteristics, but rather leads to selective progression of specific lesion dimensions. In addition, the relatively small subgroup sizes may have limited the ability to detect more subtle differences. Consequently, early and precise imaging is crucial to detect these changes and guide appropriate surgical decision-making, especially in these high-risk patients.

However, an unexpected finding emerged regarding the RHSL, with recurrent posterior dislocations demonstrating a significantly lower γ-angle (*p* = 0.003). This observation should be interpreted with caution, as only three recurrent posterior dislocations with available imaging were included in the analysis. Consequently, the finding may be influenced by limited statistical power, sample variability, or measurement uncertainty. While the difference reached statistical significance, the small sample size precludes any firm conclusions regarding its clinical relevance or underlying pathomechanisms. Nevertheless, the finding is noteworthy and may warrant further investigation in larger cohorts of patients with epilepsy-related posterior shoulder instability.

Managing shoulder injuries in epilepsy patients poses significant challenges due to a high rate of complications; in fact, instability recurs in almost two-thirds of patients after stabilization surgeries [1]. In the present cohort, 62.5% of shoulder injuries required surgical treatment, and almost half of all injuries were associated with proximal humerus fractures. These findings underscore the substantial injury burden associated with seizure-related shoulder instability. However, postoperative outcomes and recurrence rates were not consistently available in the current study; therefore, the effectiveness of the reported treatment strategies cannot be evaluated. Furthermore, 45% of patients in our cohort experienced a seizure despite existing anticonvulsant therapy. This finding highlights the complexity of seizure control in this patient population and underscores the importance of close interdisciplinary collaboration between orthopedic surgeons and neurologists throughout patient management [6].

This study has several limitations. First, the retrospective design inherently limits control over data completeness and introduces potential biases. Second, incomplete digital records limited the ability to assess these cases in detail. Radiological measurements could not be performed in all cases, as post-reduction imaging was often carried out in local hospitals and not consistently available for analysis. Furthermore, postoperative outcomes, recurrence rates, and long-term sequelae such as glenohumeral osteoarthritis were not consistently available for the entire cohort, precluding a reliable assessment of treatment efficacy and long-term clinical outcomes. In addition, information regarding adherence to anticonvulsant medication or potential discontinuation of therapy at the time of seizure was not consistently documented and therefore could not be evaluated. A more in-depth assessment of glenoid and humeral morphological changes in this patient population should be performed in future studies. Third, the study included both CT and MRI imaging for radiological assessment. Although both MRI [31–33] have been shown to be suitable modalities for measuring glenoid and humeral bone loss, the direct comparability between CT and MRI may represent a limitation. Fourth, the significant differences in radiological measurements between subgroups were relatively small, which could raise concerns about measurement error; however, the high inter- and intraobserver reliability of the measurements conducted in the present study makes this unlikely to have significantly influenced the results. Fifth, the absence of a control group of non-epileptic patients with shoulder injuries restricted the ability to directly compare injury patterns and clinical outcomes. Sixth, variations in treatment protocols and surgeon preferences over the study period may have influenced both management strategies and outcomes, thereby limiting the generalizability of the findings.

## Conclusion

In patients with epilepsy, shoulder dislocations most commonly occur during generalized seizures, with anteroinferior dislocations being the predominant direction of instability. However, posterior dislocations are considerably more common than in the general population. Although mean glenoid defect sizes were relatively small, recurrent dislocations were associated with significantly larger humeral and posterior glenoid defects, suggesting progressive osseous damage with recurrent instability. Furthermore, approximately half of all injuries were associated with proximal humerus fractures, and more than half required surgical treatment, highlighting the substantial injury burden in this patient population.

## Data Availability

All data generated or analysed during this study are included in this published article.

## Abbreviations

CI: Confidence interval
CT: Computer tomography
HSL: Hill-Sachs lesion
ICC: Intra-class correlation coefficient
MRI: Magnetic resonance imaging
RHSL: Reverse Hill-Sachs lesion
ORIF: Open reduction and internal fixation
